# Grade Classification of Tumors from Brain Magnetic Resonance Images Using a Deep Learning Technique

**DOI:** 10.3390/diagnostics13061153

**Published:** 2023-03-17

**Authors:** Saravanan Srinivasan, Prabin Selvestar Mercy Bai, Sandeep Kumar Mathivanan, Venkatesan Muthukumaran, Jyothi Chinna Babu, Lucia Vilcekova

**Affiliations:** 1 Department of Computer Science and Engineering, Vel Tech Rangarajan Dr. Sagunthala R&D Institute of Science and Technology, Chennai 600062, India; 2 School of Computer Science and Engineering, Vellore Institute of Technology, Vellore 632014, India; 3 School of Information Technology and Engineering, Vellore Institute of Technology, Vellore 632014, India; 4 Department of Mathematics, College of Engineering and Technology, SRM Institute of Science and Technology, Kattankulathur 603203, India; 5 Department of Electronics and Communications Engineering, Annamacharya Institute of Technology and Sciences, Rajampet 516126, India; 6 Faculty of Management, Comenius University Bratislava, Odbojarov 10, 820 05 Bratislava, Slovakia

**Keywords:** local-binary grey level co-occurrence matrix, enhanced fuzzy c-means clustering, convolution recurrent neural network, magnetic resonance image, image classification

## Abstract

To improve the accuracy of tumor identification, it is necessary to develop a reliable automated diagnostic method. In order to precisely categorize brain tumors, researchers developed a variety of segmentation algorithms. Segmentation of brain images is generally recognized as one of the most challenging tasks in medical image processing. In this article, a novel automated detection and classification method was proposed. The proposed approach consisted of many phases, including pre-processing MRI images, segmenting images, extracting features, and classifying images. During the pre-processing portion of an MRI scan, an adaptive filter was utilized to eliminate background noise. For feature extraction, the local-binary grey level co-occurrence matrix (LBGLCM) was used, and for image segmentation, enhanced fuzzy c-means clustering (EFCMC) was used. After extracting the scan features, we used a deep learning model to classify MRI images into two groups: glioma and normal. The classifications were created using a convolutional recurrent neural network (CRNN). The proposed technique improved brain image classification from a defined input dataset. MRI scans from the REMBRANDT dataset, which consisted of 620 testing and 2480 training sets, were used for the research. The data demonstrate that the newly proposed method outperformed its predecessors. The proposed CRNN strategy was compared against BP, U-Net, and ResNet, which are three of the most prevalent classification approaches currently being used. For brain tumor classification, the proposed system outcomes were 98.17% accuracy, 91.34% specificity, and 98.79% sensitivity.

## 1. Introduction

**Figure 1:**
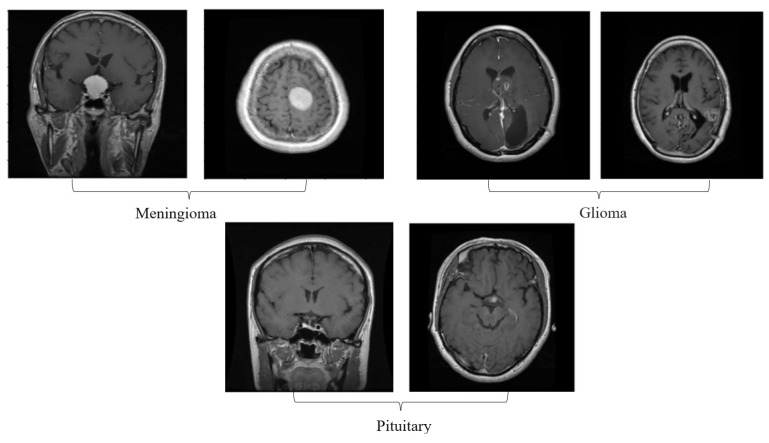
Different kinds of brain tumors based on their stage: meningioma, glioma, and pituitary.

One of the crucial tasks in medical image processing is segmenting a brain tumor. Early diagnosis of brain tumors is thought to play a crucial role in improving treatment prospects and increasing patient survival rates. The most popular technique for diagnosing tumors is magnetic resonance imaging (MRI). Additionally, since contrast-enhanced MRI provides precise information about the tumors, current research aims to enhance the MRI diagnosis by including contrast agents. Images from computed tomography (CT) also show the internal structure of organs. The involvement of radiotherapists and their expertise is necessary for the manual segmentation of tumors. Due to the vast amount of MRI (magnetic resonance imaging) data, it might result in some errors. It is a demanding and challenging task. The conditions for automatic brain tumor segmentation were thus created. Currently, machine learning methods are crucial for analyzing medical imaging [1]. An unnatural cell growth inside the skull is a brain tumor. A malignant brain tumor, which is the most dangerous type of cancer, has immediate side effects, such as decreased life expectancy and cognitive decline. Magnetic resonance imaging (MRI) analysis is a common method for finding brain tumors. In this study, we employed these images to train our novel hybrid paradigm, which combines a convolutional neural network and a neural autoregressive distribution estimation [2]. Implementing interactive computational systems is the main goal of human–computer interaction (HCI) and related technologies. The studies in HCI place a strong emphasis on the use of systems, the development of novel methods that support user activities, information access, and seamless communication. Many different domains have made extensive use of AI and deep-learning-based models, producing cutting-edge outcomes. In the current study, a model for gesture recognition relevant to the HCI domain was implemented using convolutional neural networks based on a crow search [3]. In IoT-based healthcare systems, the classification of brain tumors (BT) is crucial for the diagnosis of brain cancer (BC). The most common application of artificial intelligence (AI) methods based on computer-aided diagnostic systems (CADS) is the precise detection of brain cancer. However, because artificial diagnostic systems are inaccurate, healthcare providers are not effectively utilizing them in the diagnosis of brain cancer. In this study, we proposed a robust deep learning (DL) brain tumor classification method to address the issue of accuracy in current artificial diagnosis systems [4]. In today’s health system, clinical diagnosis has taken on a significant role. Brain cancer, which is the most serious illness and the main cause of death worldwide, is a significant area of study in the field of medical imaging. A rapid and accurate diagnosis based on magnetic resonance imaging can enhance the examination and prognosis of brain tumors. Medical imagery must be recognized, segmented, and classified in order for computer-aided diagnosis methods to assist radiologists in the accurate detection of brain tumors [5]. All aspects of human life have been impacted by technological advancements. For instance, technology’s use in medicine has significantly benefited human society. This study focused on using technology to help treat brain tumors, one of the most common and deadly diseases ever. According to a “brain tumor” website estimate, about 700,000 Americans have primary brain tumors, and each year another 85,000 people are added to this estimate. As a result, many people pass away each year. Artificial intelligence has helped humans and medicine to find a solution to this issue. The most common technique for diagnosing brain tumors is magnetic resonance imaging (MRI) [6]. Transfer learning is the name given to machine learning methods that concentrate on learning from similar tasks in order to enhance generalization in the tasks of interest. Transfer learning is crucial for developing strategies in magnetic resonance imaging (MRI) that address the variations in MR images from various imaging protocols or scanners. In addition, machine learning models that were trained to solve various tasks related to the task of interest can be used again thanks to transfer learning [7]. Researchers are required to automate brain tumor detection and diagnosis due to a sharp rise in cases. Due to the various characteristics of tumors, the classification of multiple tumors in brain images is a current research topic. Deep neural networks are now frequently used to help neurologists classify medical images. The drawbacks of deep networks include overfitting and the disappearing gradient problem [8]. The field of artificial intelligence is rapidly developing, with new opportunities in diagnostic radiology being made possible by contemporary technological advancements and the expansion of electronic health data. In the past few years, deep learning (DL) algorithms consistently got better at a wide range of medical image tasks. On non-contrast computed tomography (NCCT) of the head, DL algorithms were suggested as a tool to identify different types of intracranial hemorrhage. The capacity for DL algorithm image interpretation support might enhance the diagnostic yield of CT for identifying this urgent condition in subtle, acute cases, potentially accelerating treatment when necessary, and improving patient outcomes [9]. Because of the complexity and importance of brain tumor segmentation in the medical context, numerous automated and semi-automated segmentation processes were developed. To identify a typical medical imaging dataset, the majority of these algorithms were tested on fewer metrics with varying parameters. The various types of brain tumors shown in Figure 1 were classified according to their stage: meningioma, glioma, and pituitary [10]. A specific type of brain tumor known as a glioma can develop in a different location and have a different appearance and size. Compared with a low-grade glioma, a high-grade glioma (HGG) is a serious form of cancer (LGG). These tumors necessitate manual diagnosis, which takes time and effort. As a result, in clinical settings, MRI is helpful to evaluate gliomas because it offers crucial information about tumor regions. In this study, a feature selection method based on active deep learning was proposed to segment and identify brain tumors [11]. A deep neural network-based 3D-CNN with 3D random aspirants as a fully connected layer (FCL) was used for brain tumor segmentation, which is essential for reducing false positive (FP) rates [12].

This proposed work provides a novel automated classification strategy based on an enhanced fuzzy c-means clustering (EFCMC) algorithm for segmenting MRI images to discover a superior, more efficient automated approach to identifying and classifying brain cancers than the existing methods. We employed an upgraded version of the fuzzy c-means clustering approach in combination with adaptive filter segmentation to improve the pre-processing of MRI images exposed to the conventional classical clustering strategy for basic segmentation.

Brain malignancies, such as normal and abnormal tumors, could be classified using convolutional recurrent neural networks. The remaining sections are organized as follows: Section 1 provides an introduction, and then Section 2 discusses techniques for detecting and classifying MRI images. In Section 3, we go into the details of the proposed approach and system design. The recurrent convolutional neural network that makes use of deep learning is also explained. Experimental setups using precision, recall, and specificity metrics are discussed together with the REMBRANDT dataset used in Section 4. The results and analysis are presented in Section 5. This paper concludes in Section 6.

## 2. Related Work

The various methods currently employed to identify brain tumors are covered in this section. Saravanan et al. [13] proposed that brain tumors could be automatically segmented and identified using a deep learning-based image analysis technique. This proposed a technology that is accurate and computationally effective for segmenting brain tumors. Segmentation-CNN, pre-CNN data reduction, and refining are the three parts of this method. As an exclusive alternative to conventional single- and cross-modality-based feature extraction, tumor detection, and pixel classification using CNN, the industry-specific CNN paradigm is presented. Thirumurugan et al. [14] suggested mode-wise standardization, insight convolution, instance batch normalization, bilinear up-sampling, and balanced data insertion as some of the parts of this method that improve the quality of data input. For convolution operations that make use of high-pass filters, Full-ReLU might be able to reduce the number of kernels while maintaining the level of processing quality. The seven-layer CNN’s convolution layers only employ 108 kernels and 20,308 trainable variables. To maximize the data channel density and reduce training variability, we calculated the optimal number of kernels for each layer rather than allowing the number of layers to increase substantially. Wen Liyun et al. [15] carried out extensive tests using the BRATS-2018 database to ensure the system’s high computing quality and consistency. When you roll the dice, your chances of getting a tumor enhancement, a full tumor, or a tumor core are 76.3%, 87.4%, and 76.4%, respectively. A small portion of the total flopping described in the medical literature, namely, 28.97 g, is given to each patient. The proposed system’s easy installation and obviously superior processing quality would facilitate its use in medicine. Deep CNNs were proposed for automatically segmenting brain tumors, but they have two major drawbacks: they cannot analyze lesions at different scales and lose spatial information due to recurrent pooling and striding. Regarding the first issue, we presented a three-dimensional atrous-convolution framework for feature learning as an alternative to pooling and striding. The second issue was solved by building a pyramid of atrous convolution features and connecting them to the network’s core. By incorporating contextual information, the model’s ability to distinguish between tumors of different sizes was improved. A fully connected three-dimensional conditional random area was built as part of post-processing to provide both visual and spatial partitioning of a channel’s result. Pallavitiwari et al.’s [16] uncompressed feature computations and inter-data fusion, as shown by thorough excision testing on MRI datasets, demonstrated that our method can overcome the aforementioned problems. Our solution outperforms state-of-the-art approaches on publicly accessible benchmarks, indicating that it might be readily implemented into professional medical applications. Two-dimensional images were transformed using the wavelet method. Separate elements of the two variants were subjected to a comprehensive comparison. In addition, it was applied to a similar image to compare the global quality provided by the Fourier transform approach and the wavelet transform. The Gaussian subdivision wavelet was used for this investigation because it offered a helpful comparison to the more common Fourier analysis. An ANN algorithm was built to complete the process of segmenting MRI brain images. For their multispectral features, many models, including T1, T2-weighted, and proton-density MR images, were employed to segment distinct brain regions.

Shukla et al. [17] used the recommended ANN technique to build a learning vector quantization network. The required images were trained and assessed using the brain database of the McConnell Brain Imaging Institute. To improve the computation efficiency and precision of the proposed segmentation methods, they were evaluated using phantom brain images. However, this is dependent on the geographical organization of the data; therefore, proceed with caution. A technique for describing expert knowledge models using fuzzy cognitive maps was developed. Important FCM indications used the activating Hebbian algorithm, which is a computationally smart training method, to improve their categorization abilities. Almost 100 medical products were used to verify the proposed framework. The FCM model accurately detected low-grade (90.25%) and high-grade (91.22%) brain malignancies (54/59) with a high degree of precision. In comparison to conventional algorithms, such as fuzzy decision networks and decision trees, the output of the proposed model was considerably more precise. Comparing their performance on the same kind of main data revealed that they both performed well in terms of precision but lacked memory. Ayeshayounis et al. [18] described a three-step technique for getting MR brain images without the skull. The recommended approach was implemented using morphological processing, segmentation techniques, and smoothing filtering. Using an ADF filter, we were able to remove the noise from the MR images, and an edge detector was used to identify the anatomical borders. Finally, morphological analysis was applied to the core brain image to identify pixels that correspond to brain-like tissue. The experimental results demonstrate that the proposed method for correctly segmenting the brain is effective. Unfortunately, identifying edges was a difficult and time-consuming task. A medical decision-making framework based on normal and pathological categorization and the classification of MRI images was accomplished using a hybrid architecture. The hybrid design presented consists of three distinct stages. This approach uses discrete wavelet transformations to extract information from MRI images. Principal component analysis was then employed to further simplify the MR images. Ultimately, two classifiers were used to differentiate between healthy and abnormal MR images. In this study, we compared an artificial neural network with the K-nearest neighbor method as a classifier. Chattopadhyat et al. [19] assessed the proposed approach and found that the FP-ANN classifier attained a sensitivity of 100 percent, while the k-NN classifier acquired a specificity of 90 percent. Compared with other methods, ANN had the lowest levels of sensitivity and specificity. However, upon completion of ANN training, the output values may be incomplete and have a false tolerance. It was suggested to use a probabilistic neural network with input pre-processing and image analysis methods to automate the classification of brain cancers. Several convolutional algorithms for MR brain image classification and tumor detection were developed by humans. Mechanisms that depend on human operators to categorize vast amounts of data are unfeasible. Due to the noise in the MR image, inaccurate categorizations were produced with potentially catastrophic outcomes. Artificial intelligence, fuzzy logic, and neural networks all showed their value in this regard, and the proposed PNN classifier was applied to the decision-making process in two stages: the extraction of features through principal component analysis and classification using PNN. The classification and training accuracies were used to evaluate the effectiveness of the proposed PNN classifier. The PNN was able to quickly and accurately identify malignancies, indicating its potential as a tool for this reason. Haq et al. [4] provided a comprehensive evaluation of available surveys and DL-based diagnostic methods for PD identification. This study covered the methodologies of DL-based diagnostic approaches for PD detection, including PD dataset pre-processing, feature extraction and selection, and classification. Andronicus et al. [20] used capsule neural networks (Caps-Nets) for a revolutionary sort of machine learning (ML) architecture that was recently designed to solve the shortcomings of conventional neural networks (CNNs). Caps-Nets are resistant to rotations and affine translations, which is advantageous for analyzing medical image datasets. In addition, vision transformer (ViT)-based methods were presented relatively recently to overcome the problem of long-range dependence in CNNs. Amanullah et al. [21] showed that manual visual training for image identification may result in error detection and may be circumvented by machine learning’s most popular job. In this study, the convolutional neural network (CNN) model was developed using data augmentation and image processing methods in order to categorize the brain MRI scan images as malignant or non-cancerous and identify the different kinds of brain tumors. Saikat Islam et al. [22] presented two deep learning models for the detection of both binary (normal and abnormal) and multiclass (meningioma, glioma, and pituitary) brain cancers. We used two publicly accessible datasets that contain 3064 MRI images and 152 MRI images, respectively. To construct our models, we first applied a 23-layer convolution neural network (CNN) to the first dataset, which contained a significant number of MRI images for training. Ramdas Vankdothu et al.’s [23] primary objective was to create an internet of things (IoT) computing system based on deep learning for identifying brain cancers in MRI images. This research proposed that by combining a CNN (convolutional neural network) with an LSTM (long short-term memory), CNN’s capacity to extract features may be enhanced. O. Kouli et al. [24] showed that the automated WT segmentation’s performance is comparable to that of manual segmentation, supporting clinical practice integration. The need for more research and development of automated methods in this field is highlighted by manual segmentation’s outperformance in sub-compartmental segmentation. In terms of detection and sub-compartmental segmentation, DL performed better than TML. The interpretability and generalizability of automated models require improvements in study quality and design, including external validation. Sahar Gull et al. [25] stated that for automated segmentation and classification of brain tumors, the DL-based model is suggested. The proposed framework effectively and precisely identifies brain tumors from MR images. Segmentation is used to enhance low-contrast MR images during the pre-processing and post-processing phases. Additionally, to improve the performance, features are extracted from brain MR images using deep transfer learning techniques. The classification of MR images was carried out using GoogleNet, which is a CNN architecture. Isselmou Abd El Kader et al. [26] demonstrated the heterogeneity of the MRI images and their integration with the input images using a high pass filter. To merge slices, a high median filter was applied. The edges of the output slices were highlighted, and the MR brain images from the input were smoothed. Then, because the thresholding clusters equal pixels with the input MR data, we applied the seed-growing method based on a four-connection. When using the suggested deep wavelet auto-encoder model, the segmented MR image slices provide two layers. Based on Zahid Ullah et al.’s [27] use of multiscale residual attention (MRA-UNet), we proposed a new fully automatic method for dividing up brain tumor regions. MRA-UNet takes three slices in a row as input to maintain the sequential information and uses multiscale learning in a cascading way. This lets it use the adaptive region of interest scheme to accurately separate enhanced and core tumor regions. Based on Jaeyong Kang et al. [28], we used the idea of “transfer learning” and a few pre-trained deep convolutional neural networks to pull deep features out of brain MR images. Several machine learning classifiers then look at the deep features that were pulled out. The top three deep features that do well on several machine learning classifiers were chosen and put together to make an ensemble of deep features. This ensemble is then fed into several machine learning classifiers to predict the final output. Based on Zahid Ullah et al. [29], we present a theory that the quality of the image, which can be improved during the pre-processing stage, can make a big difference in how well any statistical approach can classify things. To make our theory stronger, we first used an improved image enhancement technique. This technique has three parts: removing noise with the median filter, boosting contrast with the histogram equalization technique, and changing the image from grayscale to RGB.

Based on the research’s results, different methods of segmentation were found to exist. These included using a region of interest (RoI), extracting features, and training and testing only classification-trained classifiers. Due to inefficient segmentation and linked feature extraction, very few features were recovered, leading to poor tumor identification and classification. The classifiers used to learn the features were also inefficient.

## 3. Proposed System

This paper presents a novel deep learning-based classification technique known as convolutional recurrent neural networks to address the deficiencies of existing approaches. The proposed work was primarily concerned with categorizing brain tumors, with the goal of reducing mortality and increasing life span. The proposed work aimed to classify brain tumors in a way that is less complicated and more accurate than the existing methods. The proposed initiative aimed to simplify the categorization of brain tumors by using the proposed technique to obtain the classification accuracy. The proposed method consists of four different steps. However, MRI gives superior soft tissue contrast than CT, and it has a better ability to distinguish between fatty, liquid, muscular, and other soft tissues (CT is usually better at imaging bones). These images provide clinicians with information that could be used to diagnose a broad spectrum of diseases and disorders.

**Figure 2:**
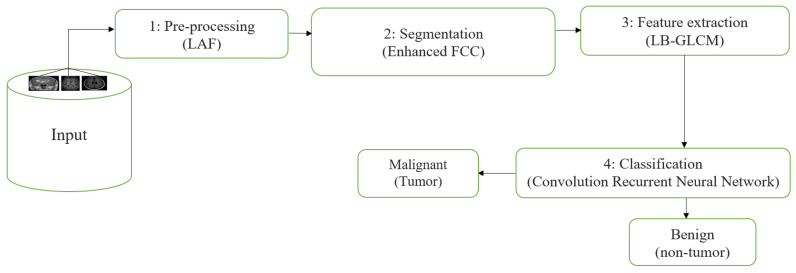
Proposed system workflow.

An adaptive filtering method is used in the initial stage of processing, and a clustering technique is used in the second step of segmentation. Third, a local-binary gray-level co-occurrence matrix is used to extract features. Classification with CRNN is the fourth and final process. Figure 2 is a thorough explanation and workflow of the proposed model. The proposed framework consists of four different stages: (i) the linear adaptive filter is used to denoise the input image as in the pre-processing stage, (ii) the enhanced fuzzy c-means clustering is used in the segmentation stage, (iii) the local-binary grey level co-occurrence matrix is used to extract the recommended feature in the feature extraction stage, and (iv) a convolutional recurrent neural network is used to classify the image in order to address the disordered images in the classification stage.

### 3.1. Stage 1: Pre-Processing—Linear Adaptive Filter

In this stage, we analyzed the images to their lowest depths and filtered them according to competing elements, all in an effort to reduce the noise and unwanted anomalies in the images before they are sent to the segmentation tools. If we filter the image using the median value or an adaptive filter method, we may get cleaner and more accurate results. Eliminating noise is a fundamental goal of image pre-processing in order to restore lost details and sharpness to a damaged image. In the case of linear adaptive filtering, denoising is carried out in accordance with the regional noise information that is already present in the image [30]. The degraded image is referred to as $\hat{K}(a,b)$, and the noisy image variance in its entirety is shown by $\alpha_{b}^{2}$. The noise variance is then estimated from the difference between the noisy image and the filtered image. The local mean is referred to as the ${\hat{\forall }}_{M}$ pixel of the window, then the ${\hat{\alpha }}_{b}^{2}$ is the variance local in a window and the noise removal from the image can be done using Equation (1), where (*a*, *b*) are the noisy images:

(1) $$\hat{K}\left(a,b\right)-\frac{\alpha_{b}^{2}}{{\hat{\alpha }}_{b}^{2}}(\hat{K}\left(a,b\right)-{\hat{\forall }}_{M})$$

Here, when the noise level is nearest to zero, then Equation (1) can be written as

(2) $$\alpha_{b}^{2}=0=>\hat{\hat{K}}=\hat{K}\left(a,b\right)$$

Global noise in an image is the presence of artifacts that do not originate from the original scene content. If the global noise variance is small, and the local variation is significant, then the ratio is equal to one, i.e.,

(3) $${{\hat{\alpha }}_{b}^{2}}^{\prime \prime }\alpha_{b}^{2},then\hat{\hat{K}}=\hat{K}\left(a,b\right)$$

A high local variance describes the appearance of an edge in the examined image window. When the local and global variations are similar, the equation becomes

(4) $$\hat{\hat{K}}={\hat{\forall }}_{M}\;as\;{\hat{\alpha }}_{b}^{2}\approx \alpha_{b}^{2}$$

In a normal area, the output is often displayed above the similarities as the average value of the window across the pixel.

**Figure 3:**
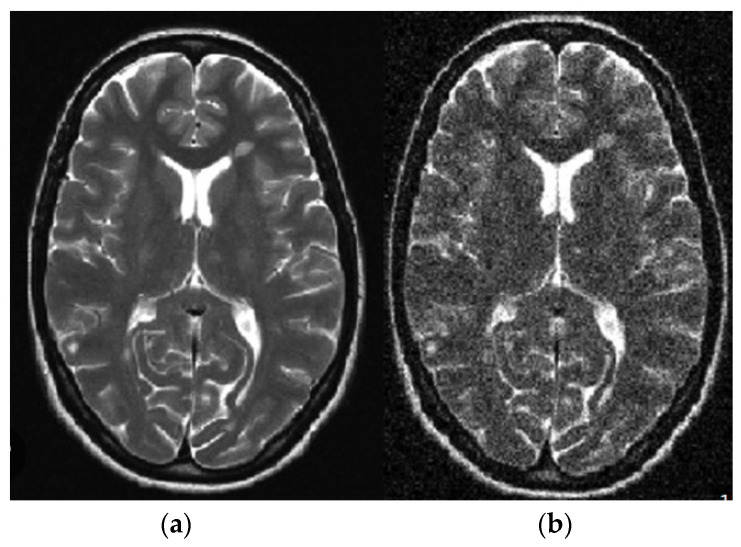
Linear adaptive filter image: (**a**) noisy; (**b**) noiseless.

For a standard application in a healthy region, the mean value output of the window throughout a pixel is simply provided above the similarities. If no anomalies are performed, an edge is transferred to the result. This is an essential function of an adaptive filter. The filter uses the window size as the input and manages the balance based on the input image [31]. The subsequent results were used to investigate noise elimination in brain tumor images using the adaptive filter shown in Figure 3. The purpose was to investigate the impact of adaptive noise removal on the edges.

### 3.2. Stage 2: Segmentation—Enhanced Fuzzy C-Means Clustering

Since the scan generates so many images, the segmentation step is crucial. Amazingly, medical professionals have to manually segregate these images in a constant way. There are several different segmentation methods that may be used for dividing up images [32]. The segmentation methods used will vary depending on the characteristics that are analyzed and extracted. The fuzzy c-means clustering method may be used to partition an image into non-overlapping sub-regions [33]. Segmentation is the process of dividing a large image into smaller, more manageable portions of pixels for the purpose of analysis. Fuzzy c-means clustering is often used to refine the image’s edges or objects; the resultant pieces form a whole. When processing images, the fuzzy c-means clustering algorithm often focuses on either the similarities or differences between them. It is useful in medical diagnostics and may aid in preoperative planning and robotic surgery [34]. Many different types of segmentation methods are feasible and applicable, such as threshold-based segmentation, region-based segmentation (region growing, splitting, and merging methods), histogram-based segmentation methods, edge-based segmentation methods, and clustering approaches [35]. Using this unsupervised clustering technique, data from one set cannot be included in any other cluster. This technique may be easily integrated into pre-existing clustering frameworks [36]. The enhanced fuzzy c-means clustering algorithm is given below (Algorithm 1).
**Algorithm 1** Enhanced Fuzzy C-MeansStart: Set values for the number of clusters d, the degree of fuzziness m > 1, and the error ε. Step 1: Input the MRI brain image Step 2: Initiate the greyscale image conversion Step 3: Apply the denoising method to a greyscale image Step 4: Initiate the image-sharpening process Step 5: Apply a linear adaptive filter in order to obtain the denoised image Step 6: Compute the fuzzy c-means segmentation Step 7: Randomly initialize the cluster centers, $d_{ci}^{\left(0\right)}$ Step 8: n←1 Step 9: Repeat Step 10: Compute the membership of the matrix $M_{m}^{\left(n\right)}$ utilizing a center $d_{ci}^{\left(n-1\right)}$,
(5)$$V_{cij}^{\left(n\right)}\leftarrow \frac{1}{\sum_{l=1}^{d}{\left(\frac{d(a_{j},d_{ci}^{\left(n-1\right)})}{d(a_{j},d_{cl}^{\left(n-1\right)})}\right)}^{\frac{2}{n-1}}}$$Step 11: *Here,*
$V_{cij}^{\left(n\right)}$ are the enhanced fuzzy c-means membership parameters Step 12: *n←n+1* Step 13: *Until*
$\left\|V_{cij}^{\left(n\right)}-V_{cij}^{\left(n-1\right)}\right\|<\in$ Step 14: Calculate the thresholding value of segmentation Step 15: Exact outcome obtained as a malignant region Stop

**Figure 4:**
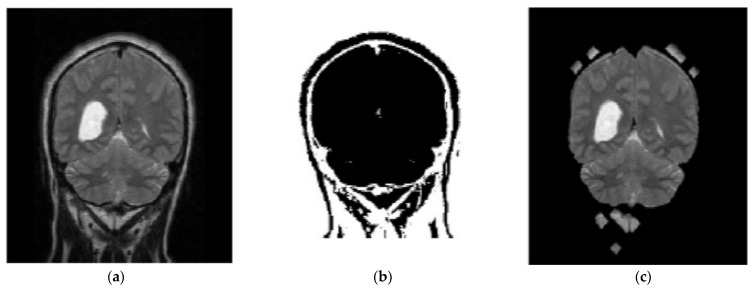
Brain MRI images: (**a**) normal; (**b**) binary; (**c**) after a morphological operation.

**Figure 5:**
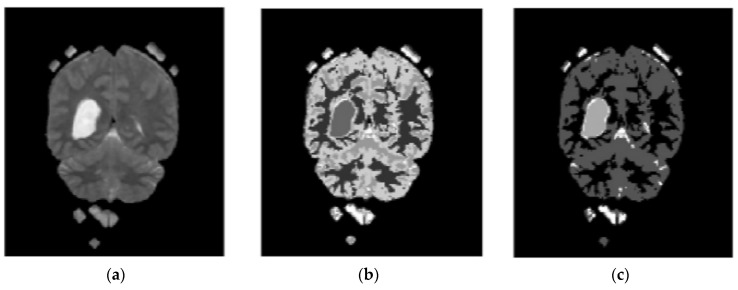
Brain images: (**a**) enhanced; (**b**) fuzzy c-means 1; (**c**) fuzzy c-means 2.

In the proposed segmentation approach, enhanced fuzzy c-means clustering is applied for brain tumor segmentation. In this method, an image’s N pixels are divided into fuzzy c clusters, where c is a positive number and n is a smaller number. Figure 4 and Figure 5 depict various sets of MRI brain images, such as the normal or original image, the binary level image, and the post-morphological operation outcome [37]. The segmentation process is taken as an enhanced version of the fuzzy c-means clustering (EFCMC) algorithm. This includes various stages, as shown in Figure 4 and Figure 5. After performing EFCMC, a final segmented tumor appeared. The proposed segmentation process took less time and was computationally less difficult than other methods.

An additional set of data was provided in the form of clusters. Alternately, we used a refined version of the fuzzy c-means clustering technique. It is useful for extracting features using LBGLCM and yields decent results when used with segmentation methods.

**Figure 6:**
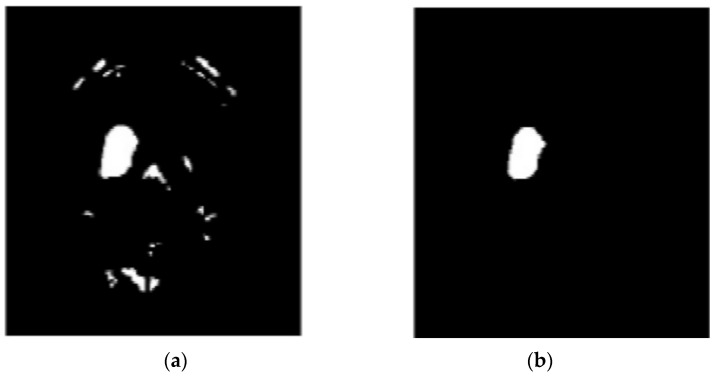
(**a**) Fuzzy c-means 3; (**b**) segmented tumor region.

A total of 620 MRI scans of the brain were analyzed, 612 of which were deemed normal and 8 of which showed signs of tumor growth (abnormal). It took a few stages of the enhanced fuzzy c-means clustering method to get the area of the brain where the tumor was located. The upgraded fuzzy c-means clustering technique was assumed to be used in the segmentation process. There were several steps involved, as seen in Figure 4, Figure 5 and Figure 6. The tumor was successfully segmented after the EFCMC. The proposed segmentation method was faster and required fewer computer resources than other methods. Figure 6 illustrates the brain MRI images, such as the third stage of the enhanced fuzzy c-means cluster impact and it provides the outcome of the segmented tumor region.

### 3.3. Stage 3: Feature Extraction—Local Binary GLCM

**Figure 7:**
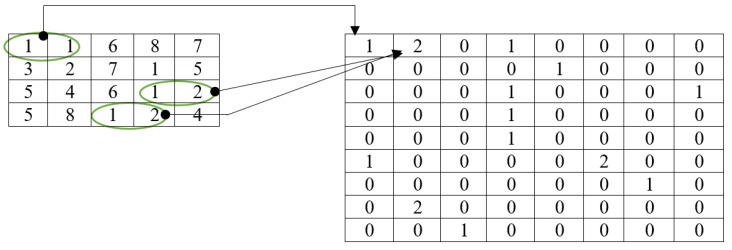
Feature extraction using LBGLCM.

This method is based on a union of the LBP and GLCM techniques. The first step of this process is running the LBP operator on the original raw image. The LBP operator examines the image for patterns in order to produce a texture map. The last step is to extract the GLCM features from the LBP image that was created. When extracting features, the conventional GLCM algorithm uses both the current pixel and its neighboring pixel [38]. It does not take other nearby patterns on the image into consideration. However, the LBGLCM approach takes into account the texture’s whole characteristics and feature space before extracting any characteristics. On account of these merits, the LBGLCM method outperformed the GLCM algorithm in several image-processing tasks. In this research, we found that the same formulae used by the GLCM algorithm to extract characteristics from histopathological images may be used in the LBGLCM method. Figure 7 depicts the feature extraction done using the LBGLCM operation.

To define an image’s texture, the LBGLCM functions determine the frequency with which pairings of pixels with a given value and a certain spatial connection occur in the image, generate an LBGLCM, and then derive statistical metrics from this matrix. The different LBGLCM features are as follows:

(6) $$Energy\left(E_{enr}\right)=>E_{enr}=\sum_{x=0}^{i-1}\sum_{y=0}^{i-1}L_{B}{\left(x,y\right)}^{2}$$

(7) $$Entropy=>E_{ent}=-\sum_{x=0}^{i-1}\sum_{y=0}^{i-1}L_{B}\left(x,y\right)ln$$

(8) $$Contrast=>Con=\sum_{x=0}^{i-1}\sum_{y=0}^{i-1}L_{B}{\left(x,y)(x-y\right)}^{2}$$

(9) $$Homogeneity=>Hom=\sum_{x=0}^{i-1}\sum_{y=0}^{i-1}\frac{L_{B}(x,y)}{\left(1+{\left(x-y\right)}^{2}\right)}$$

(10) $$Correlation=>Cor=\sum_{x=0}^{i-1}\sum_{y=0}^{i-1}\frac{L_{B}\left(x,y\right)[\left(x-\mu_{i}\right)\left(y-\mu_{j}\right)]}{\sigma_{i}\sigma_{j}}$$

The LBGLCM technique’s summing of squared element values is included in this. Regular image energies were 1, and the range for the quantity of energy was from 0 to 1. This is a calculation for determining LBGLCM values based on the proximity of items dispersed in the LBGLCM. Values of homogeneity varied from zero to one [39].

### 3.4. Stage 4: Classification—Convolution Recurrent Neural Network

**Figure 8:**
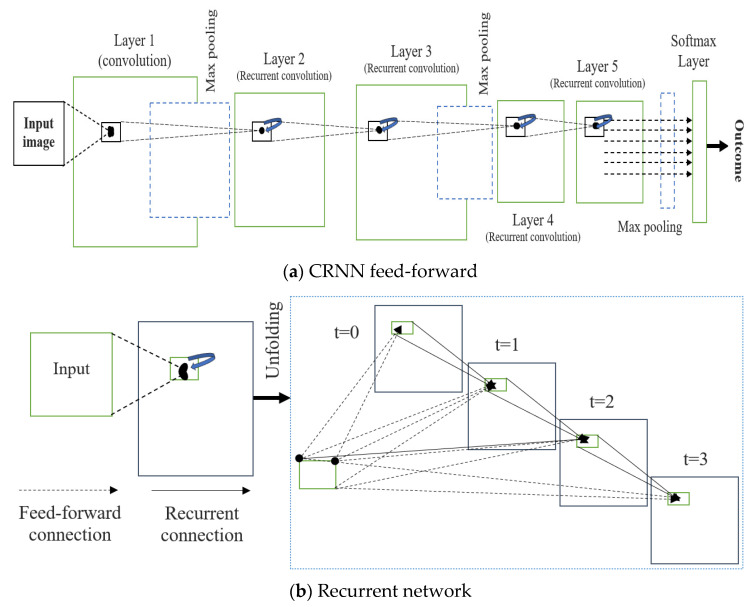
Proposed CRNN system architecture. (**a**) Feed-forward; (**b**) Recurrent.

Each image was put through a classification process to determine its proper categorization based on its characteristics. Imaging of the brain may be difficult to interpret, and thus, classification is a crucial tool for distinguishing between normal and tumorous regions. In data mining, classification is a process that assigns values to elements so that they may be used as criteria for other operations [18]. The goal of the classification is to reliably forecast the final class for each data occurrence. Following the CNN layer with the advanced CRNN layer and replacing the fully connected layer with the CNN layer would allow the present image classification algorithm to learn MR brain image measurements. Figure 8 depicts the operation workflow of the proposed CRNN architecture. Active use of RCNN for the categorization of brain tumors was the key motivation for this study. CNN results in this area have not improved much in the previous two years, but RNNs can include CNNs because of the stronger positive connection of contextual data. CNNs already perform better than CRNNs when classifying medical images, but CRNNs have the potential to catch up. RNNs are suitable for handling temporal or sequential data, sparsity, and reusing the same neurons and weights over time. However, conventional techniques are suitable for handling spatial data (images) and reusing the same neurons over different parts of the image. Finally, the RCNN model is the hybrid model that handles both the sequential and spatial dataset information and reuses the same neurons over time (weight) and various portions of the image.

The convolutional recurrent layer (CRL) is the backbone of a convolution recurrent neural network (CRNN). Over the course of a single time interval, the CRL components evolve:

(11) $${\left(\left(v_{o}^{e}\right)\left(v_{o}^{e}\right)\left(v_{o}^{e}\right)\right)}^{T}{\left(w^{\left(i,n\right)}\left(t\right)+v_{o}^{s}\right)}^{T}y^{\left(i,n\right)}\left(t-1\right)+c_{o}$$

Here, $w^{\left(i,n\right)}$ is referred to as a feed-forward and $y^{\left(i,n\right)}\left(t-1\right)$ is an input of the recurrent, where these are vectorized points aligned at *(i*, *n)* of the present layer feature map. Here, $v_{o}^{e}$ are the weights of the feedforward, $v_{o}^{s}$ is a weight of the recurrent, and $c_{o}$ is the bias. Equation (11) represents the two phrases of connections, such as between a traditional CNN and a recurrent network.

(12) $$y_{ino}=h(e\left(y_{ino}\left(t\right)\right))$$

Here, *e* refers to the modified linear function call activation and is expressed in Equation (13), where *h* refers to the response local normalization and can be written as

(13) $$e\left(a_{ino}\left(t\right)\right)=maximum(M_{ino}\left(t\right),0)$$

(14) $$h(e_{ino}\left(t\right))=\frac{e_{ino}\left(t\right)}{{\left\{\left(e_{ino^{\prime }}\right){\left(e_{ino^{\prime }}\right)}^{2}\right\}}^{\beta }}$$

where K is the current layer’s maximum number of feature maps. In Equation (14), and are constants that govern the normalization, and N is the number of feature maps, with the sum running at the same point of (*i*, *n*) over all N. The cortex, where many aspects obtain extensive responses, is influenced by RLN’s ability to inhibit lateralization. In our scheme, we used RLN to prevent states from simultaneously being shown. Figure 6 shows CRL’s step length for T = 3 s, indicating a feedforward subnet with a major depth of 4, a minor depth of 1, and a medium depth of 2. Only the feedforward computation needs a location at t = 0. One convolutional layer, four convolutional regularization layers, three max-pooling layers, and one softmax layer make up the CRNN used here on the right side of the image. Furthermore, RCNN mixes a max-pooling CRL layer stack with interleaved layers. The longest route makes use of all frequently open connections (thus the duration = T + 1), while the shortest route takes advantage of the more convenient F.F. link (hence the length = 1). Table 1 illustrates the proposed CRNN layer structure.

## 4. Experimental Setup

**Figure 9:**
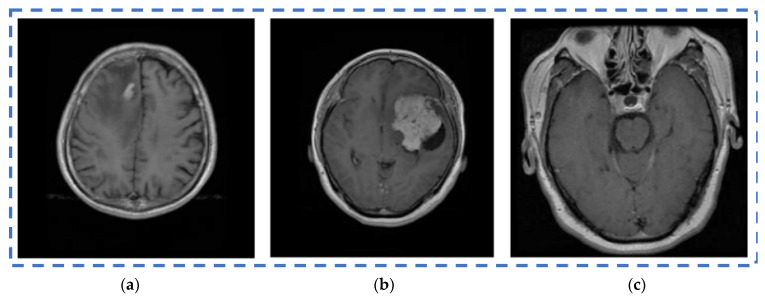
Different types of tumors in the brain region from the REMBRANDT dataset. (**a**) Meningioma; (**b**) Glioma; (**c**) Pituitary.

The REMBRANDT publicly available dataset, which is a publicly available database, was used to test the proposed method. The data set was partitioned into two parts: training and testing. Images of glioma tumors, meningioma tumors, pituitary tumors, and no tumors were included in both the training and testing sets. A total of 3100 images are taken, and the image dataset percentages for training and testing the proposed system framework were 80% and 20%, respectively. Thus, both a training set of 2480 images and a testing set of 620 images were used, and data pre-processing operations, such as brain stripping, were offered to improve the data description [40]. However, the 3100 images were like a combination of different grade (tumor) images in order to fine-tune the proposed model to obtain a better classification accuracy [41]. The performance assessment work was divided into four groups: glioma tumor, meningioma tumor, no tumor, and pituitary tumor. Figure 9 displays many instances of various tumors: (a) meningioma, (b) glioma, and (c) pituitary. Table 2 illustrates the total number of images that were used in the proposed system during the training and testing phases.

The proposed work was implemented in Python’s Keras package, which is geared toward machine learning. For the purpose of implementing neural networks, Python is an advanced programming language that may be used in conjunction with TensorFlow. This aids the central processing unit and graphics processing unit performance. To fine-tune the hyperparameters, we first did a network discovery and then used those parameters with which the plan performed best on the selected test data. There were variations in test conditions, such as testing pace and energy. Starting with an energy level of 0.5, it was gradually increased to 0.9, and the learning rate was adjusted from 0.002 to 0.3 × 10^5^ over the course of many iterations. Accuracy, specificity, and sensitivity were used to approximate the research observations. True positive, true negative, false positive, and false negative values were used to determine these standards.

### 4.1. Experimental Results

The accuracy, sensitivity, and specificity were used to estimate the results of the experiment. The true positive (*TP*), true negative (*TN*), false positive (*FP*), and false negative (*FN*) values were used to figure out these measurements. In the following equations, you can see how the evaluation measures work. The detection accuracy of brain tumors can be measured using the ratio of the population’s exact values. The following formula describes the accuracy:

(15) $$Accuracy=\frac{TP+TN}{TP+FP+FN+TN}$$

The sensitivity for detecting brain tumors is defined by the ratio of true positives to the false negatives and true positives. This relationship can be stated as follows:

(16) $$Sensitivity=\frac{TP}{TP+FN}$$

Using the ratio of true negatives to the false positives and true negatives, the specificity of brain tumor detection can be determined. It can be portrayed as

(17) $$Specificity=\frac{TN}{TN+FP}$$

**Figure 10:**
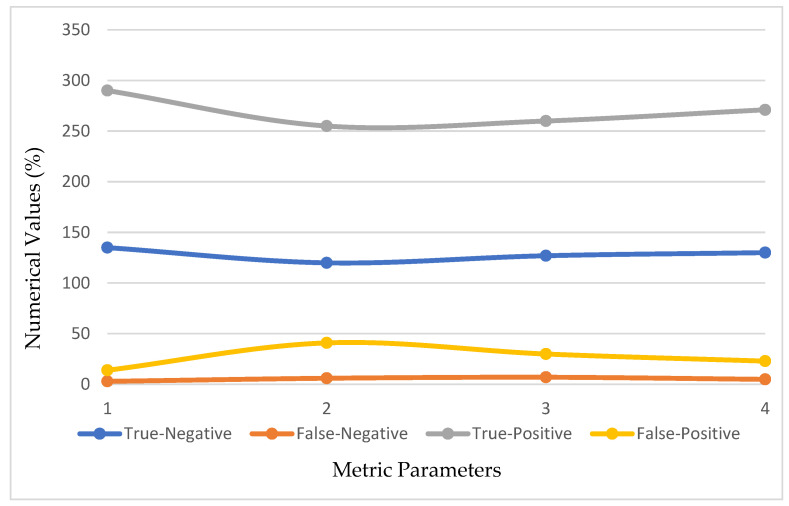
Evaluation metric index outcomes of the CRNN and other classification methods.

**Figure 11:**
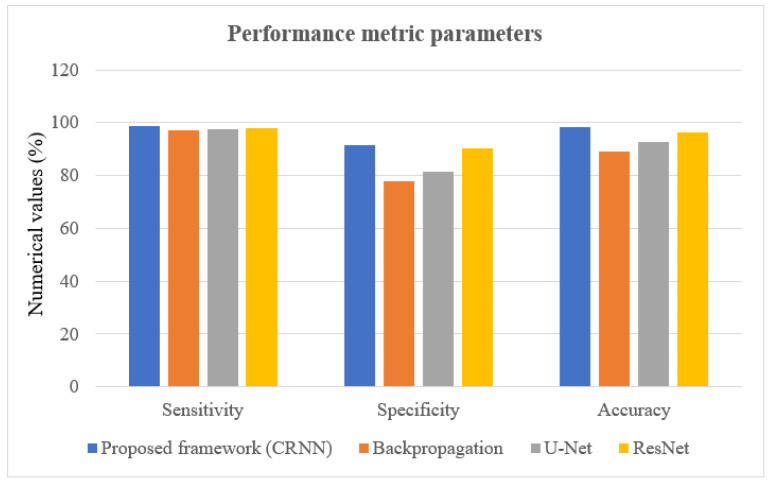
Performance metric comparison graphical representation.

**Figure 12:**
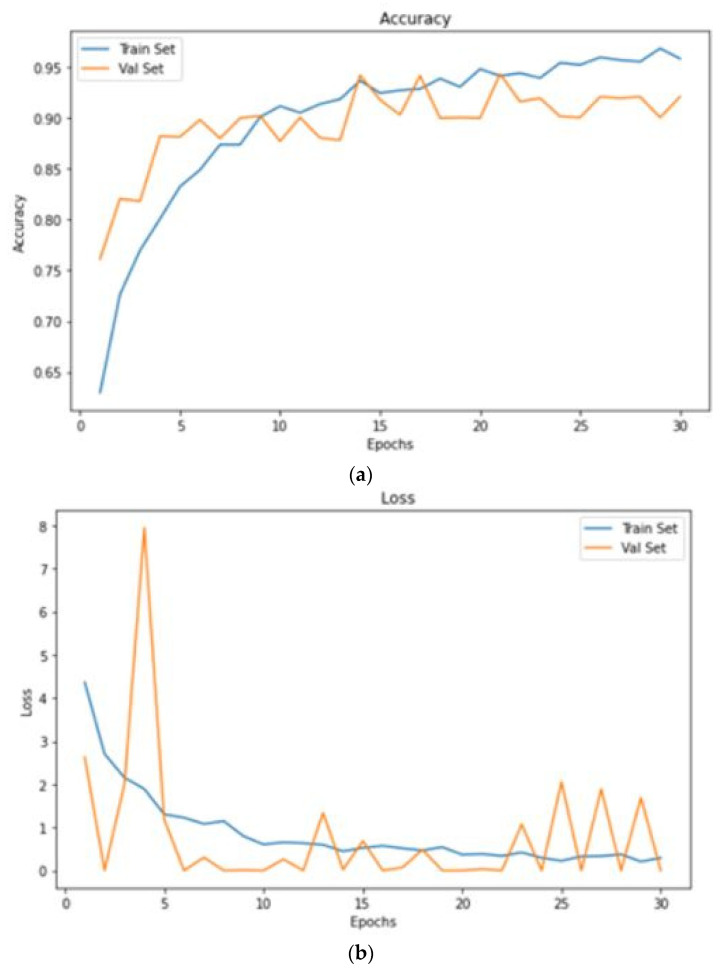
Proposed model training and validation accuracy with 30 epochs. (**a**) accuracy; (**b**) loss.

In this portion, we describe the results of applying the proposed approach to the publicly available dataset and analyzing and computing its results. A total of 620 MR images were used for testing, whereas 2480 MR images from 235 patients were used for training. The prediction model with low variation in energy levels of a comparable class that was straightforward to categorize was also included in the dataset. As a result, the proposed model was estimated using only reliable patient data. The metrics for assessment were specified for four different tumor locations: (a) glioma tumor, (b) meningioma tumor, (c) no tumor, and (d) pituitary tumor. The experimental results and a representative image are shown in Table 3. The total number of utilized images from the dataset was 620, and each image was classified as either normal (284) or malignant (336). In order to evaluate the efficacy of the proposed model, we compared it with other classifiers in Table 4. The sensitivity, specificity, and accuracy were compared across several methods in Table 5. There is an evaluation of the proposed CRNN classifier against the BP, U-Net, and CRNN methods. Table 6 depicts the proposed CRNN and other classifier evaluation parameters comparison outcomes. Figure 10 depicts the relative accuracy (in percent) of several categorization methods along an X-axis representing the methods themselves. A graph depicting the comparison of algorithms used by BP, U-Net, ResNet, and CRNN classification methods is given. The other methods of categorization produced the following results: backpropagation [34] obtained a Se of 97.11%, Sp of 77.71%, and Acc of 89.12%; the U-Net [35] obtained the outcomes of an Se of 97.56%, Sp of 81.51%, and Acc of 92.61%; and the ResNet [36] model achieved an Se of 97.9%, Sp of 90.23%, and Acc of 96.23%. The proposed CRNN showed substantial improvement in the parameter outcomes, namely, an Acc of 98.17%, Se of 91.34%, and Sp of 98.79%. Figure 11 depicts Performance metric comparison graphical representation. Figure 12 depicts the proposed model training and validation accuracy with 30 epochs.

### 4.2. Ablation Studies

The Python-based Keras library for machine learning was used to implement the suggested solution. Python is a high-level programming language that may be used with TensorFlow to create neural networks. This facilitates processing on both the CPU and GPU. By using a network search and the parameters on which the plan performed the best on the selected test data, the hyperparameters are tweaked. When testing, variables such as the testing rate and energy were different.

## 5. Conclusions

The proposed work contained pre-processing, segmentation, MR image feature extraction, and classification. Enhanced fuzzy c-means clustering and a trained deep learning convolutional neural network (DCNN) were used to divide the brain tumor images into different parts. Here, 620 test images and 2480 training images were utilized with the Python Keras package to evaluate the efficacy of the proposed technique on the REMBRANDT dataset. In addition to backpropagation (BP), U-Net, ResNet, and RCNN, the CRNN classification strategy was assessed alongside other neural network-based classification algorithms. The results represent that the MRI scan can be used to identify the tumors more accurately than existing approaches. The proposed CRNN strategy was compared with BP, U-Net, and ResNet, which are three of the most prevalent classification approaches. The proposed brain tumor classification obtained better outcomes, namely, 98.17%, 91.34%, and 98.79% for accuracy, specificity, and sensitivity, respectively.

## 6. Future Work

We have a plan to start a deep investigation into different medical image modalities in the future. In order to undertake a knowledge-driven NN search for brain tumor detection, we plan to include more medical information in our future paradigm. This includes a clinical guideline for MRI-based diagnosis and grade evaluation for brain tumors. We want to expand our paradigm for multi-modal brain tumor categorization by using other modalities, such as diagnostic processes, health information, and respiratory rates, in addition to MRI images.

## Figures and Tables

**Table 1:** The layer structure of the proposed CRNN.

Name of Layer	Layer Properties		
Input layer	64 × 64 × 3	-	-
Convolution layer	256—number of filters	3 × 3—size of the filter	1 × 1—stride
Activation layer (ReLU)	-	-	-
Convolution layer	256—number of filters	3 × 3—size of the filter	1 × 1—stride
Activation layer (ReLU)	-	-	-
Pooling layer	Max-pooling	2 × 2—size of the filter	2 × 2—stride
Convolution layer	256—number of filters	3 × 3—size of the filter	1 × 1—stride
Activation layer (ReLU)	-	-	-
Convolution layer	256—number of filters	3 × 3—size of the filter	1 × 1—stride
Activation layer (ReLU)	-	-	-
Pooling layer	Max-pooling	2 × 2—size of the filter	2 × 2—stride
Fully connected layer (512)			
Activation layer (ReLU)			
Dropout layer (0, 5)			
Fully connected layer (2)			
Softmax layer			
Classification layer			

**Table 2:** Image utilization from the dataset.

Data Set Split-Up	
Total no. of images (A)	3100
Training images (80% of A)	2480
Testing images (20% of A)	620

**Table 3:** Experimental study of several photos showed that the proposed classifier recognized glioma, normal, meningioma, and pituitary brain cancers.

Input (Image)	Segmented Image	Tumor Type	Tumor-Affected Portion	Total Count of Defective Cells
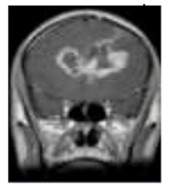	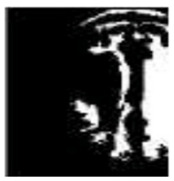	Glioma	28.521	18,112
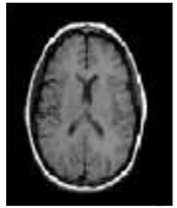	Not required for benign images.	Benign	0	0
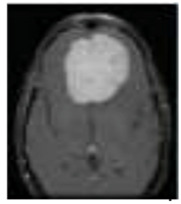	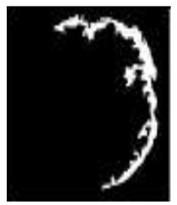	Meningioma	19.131	4951
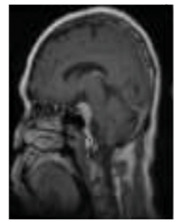	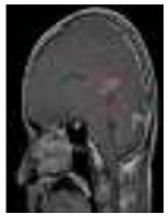	Pituitary	23.1910	5361

**Table 4:** Proposed CRNN evaluation parameters comparison with other classification techniques.

Evaluation Parameters	Proposed Framework (CRNN)	Backpropagation [31]	U-Net [32]	ResNet [33]
Sensitivity (Se)	98.79	97.11	97.56	97.9
Specificity (Sp)	91.34	77.71	81.51	90.23
Accuracy (Acc)	98.17	89.12	92.61	96.23

**Table 5:** Evaluation metric index of proposed and other classifier techniques.

Performance Metric Indexes	Proposed Framework (CRNN)	Backpropagation	U-Net	ResNet
True negative	135	120	127	130
False negative	3	6	7	5
True positive	290	255	260	271
False positive	14	41	30	23

**Table 6:** Proposed CRNN performance metric comparison with other classification methods.

Performance Metric Parameters (%)	Proposed Framework (CRNN)	Backpropagation	U-Net	ResNet
Sensitivity	98.79	97.11	97.56	97.9
Specificity	91.34	77.71	81.51	90.23
Accuracy	98.17	89.12	92.61	96.23

## Data Availability

Not applicable.
